# Supplementary material for: The Systems Biology Research Tool: evolvable open-source software
Source: BMC Syst Biol. 2008 Jun 29;2:55. doi: 10.1186/1752-0509-2-55 (PMC2446383; doi:10.1186/1752-0509-2-55)
Supplement: Additional file 1 — SBRT Archive. An archive of the current version of the Systems Biology Research Tool. [file 1752-0509-2-55-S1.zip › sbrt-1.4.0/doc/developers_guide/api/sbrt/kernel/fba/proc/RandomEnvGeneratorV1.html]

RandomEnvGeneratorV1


|  |  |  |
| --- | --- | --- |
| **Overview** \| **Package** \| **Class** \| **Use** \| **Tree** \| **Deprecated** \| **Index** \| **Help** | |  |
| **PREV CLASS**   **NEXT CLASS** | **FRAMES**    **NO FRAMES**     **All Classes** |  |
| SUMMARY: NESTED \| FIELD \| CONSTR \| METHOD | DETAIL: FIELD \| CONSTR \| METHOD |  |


---


## sbrt.kernel.fba.proc Class RandomEnvGeneratorV1

```
java.lang.Object
  sbrt.kernel.fba.proc.FbaProcess<java.util.Map<java.lang.String,Interval>>
      sbrt.kernel.fba.proc.RandomEnvGeneratorV1
```

**All Implemented Interfaces:**: KernelProcess<java.util.Map<java.lang.String,Interval>>, MonitoredProcess<java.util.Map<java.lang.String,Interval>>

---

``` public final class RandomEnvGeneratorV1 extends FbaProcess<java.util.Map<java.lang.String,Interval>> implements MonitoredProcess<java.util.Map<java.lang.String,Interval>> ```

This class is used to generate random environments for use in flux balance
analysis.

**Author:**
:   This class was written and documented by Jeremiah Wright while in the
    Wagner lab.

---

**Constructor Summary**

`RandomEnvGeneratorV1(IrrevRxnFluxome fluxome)`
  
          Constructs a new random environment generator.


| **Method Summary** | |
| --- | --- |
| `Interval` | `getActive()`             Returns the constraint used to represent an active source flux. |
| `int` | `getActiveFluxes()`             Returns the number of active source fluxes in each randomly generated environment. |
| `IrrevRxnFluxome` | `getFluxome()`             Returns the fluxome for which random objective functions are to be generated. |
| `Interval` | `getInactive()`             Returns the constraint used to represent an inactive source flux. |
| `int` | `getIterations()`             Returns the number of random environments to be generated. |
| `ProgressMonitor` | `getProgress()`             Returns a progress monitor for this process. |
| `void` | `run(ResultsManager<java.util.Map<java.lang.String,Interval>> resultsManager)`             Generates a random environment and adds it to the provided results manager. |
| `void` | `setActiveFluxes(int activeFluxes)`             Sets the total number of active source fluxes to be contained in each randomly generated environment. |
| `void` | `setIterations(int iterations)`             Sets the number of random environments to be produced. |
| `void` | `setOff(Interval inactive)`             Sets the constraint used to represent an inactive source flux. |
| `void` | `setOn(Interval active)`             Sets the constraint used to represent an active source flux. |
| `void` | `setRandom(java.util.Random random)`             Sets the random number generator to the one provided. |

**Methods inherited from class sbrt.kernel.fba.proc.FbaProcess**

`toString`

**Methods inherited from class java.lang.Object**

`clone, equals, finalize, getClass, hashCode, notify, notifyAll, wait, wait, wait`

**Constructor Detail**

### RandomEnvGeneratorV1

```
public RandomEnvGeneratorV1(IrrevRxnFluxome fluxome)
```

:   Constructs a new random environment generator.

    **Parameters:**: `fluxome` - the fluxome for which random environments are to be generated.


**Method Detail**

### getFluxome

```
public IrrevRxnFluxome getFluxome()
```

:   Returns the fluxome for which random objective functions are to be
    generated.

    :   **Specified by:**: `getFluxome` in class `FbaProcess<java.util.Map<java.lang.String,Interval>>`
    :   **Returns:**: the fluxome for which random objective functions are to be generated.

---


### getIterations

```
public int getIterations()
```

:   Returns the number of random environments to be generated.

    :   **Returns:**: the number of random environments to be generated.

---


### setIterations

```
public void setIterations(int iterations)
```

:   Sets the number of random environments to be produced.

    :   **Parameters:**: `iterations` - the number of random environments to be produced.

---


### getActiveFluxes

```
public int getActiveFluxes()
```

:   Returns the number of active source fluxes in each randomly generated
    environment.

    :   **Returns:**: the number of active source fluxes in each randomly generated environment.

---


### setActiveFluxes

```
public void setActiveFluxes(int activeFluxes)
```

:   Sets the total number of active source fluxes to be contained in each
    randomly generated environment.

    :   **Parameters:**: `activeFluxes` - the total number of active source fluxes to be contained in each randomly generated environment.

---


### setRandom

```
public void setRandom(java.util.Random random)
```

:   Sets the random number generator to the one provided.

    :   **Parameters:**: `random` - the random number generator to use.

---


### getActive

```
public Interval getActive()
```

:   Returns the constraint used to represent an active source flux.

    :   **Returns:**: the constraint used to represent an active source flux.

---


### getInactive

```
public Interval getInactive()
```

:   Returns the constraint used to represent an inactive source flux.

    :   **Returns:**: the constraint used to represent an inactive source flux.

---


### setOn

```
public void setOn(Interval active)
```

:   Sets the constraint used to represent an active source flux.

    :   **Parameters:**: `active` - the constraint used to represent an active source flux.

---


### setOff

```
public void setOff(Interval inactive)
```

:   Sets the constraint used to represent an inactive source flux.

    :   **Parameters:**: `inactive` - the constraint used to represent an inactive source flux.

---


### getProgress

```
public ProgressMonitor getProgress()
```

:   Returns a progress monitor for this process.

    :   **Specified by:**: `getProgress` in interface `MonitoredProcess<java.util.Map<java.lang.String,Interval>>`
    :   **Returns:**: a progress monitor for this process.

---


### run

```
public void run(ResultsManager<java.util.Map<java.lang.String,Interval>> resultsManager)
```

:   Generates a random environment and adds it to the provided
    results manager. Each random environment contains a constant
    number of active and inactive constraints. It is the assignment of these
    constraints to reaction names that is random. For each metabolite source that
    is set to be active, its corresponding metabolite sink reaction is constrained
    to the interval [0, 0].

    :   **Specified by:**: `run` in interface `KernelProcess<java.util.Map<java.lang.String,Interval>>`
    :   **Parameters:**: `resultsManager` - the results manager to which reaction names mapped to their randomly assigned constraint is added.


---


|  |  |  |
| --- | --- | --- |
| **Overview** \| **Package** \| **Class** \| **Use** \| **Tree** \| **Deprecated** \| **Index** \| **Help** | |  |
| **PREV CLASS**   **NEXT CLASS** | **FRAMES**    **NO FRAMES**     **All Classes** |  |
| SUMMARY: NESTED \| FIELD \| CONSTR \| METHOD | DETAIL: FIELD \| CONSTR \| METHOD |  |


---
